# Metformin ameliorates insulitis in STZ-induced diabetic mice

**DOI:** 10.7717/peerj.3155

**Published:** 2017-04-13

**Authors:** Xue Han, Yu-Long Tao, Ya-Ping Deng, Jia-Wen Yu, Jian Cai, Guo-Fei Ren, Yuan-Nan Sun, Guo-Jun Jiang

**Affiliations:** 1 Department of Pharmacy, Xiaoshan Hospital, Hangzhou, China; 2 Department of Pharmacy, Changzheng Hospital, Second Military Medical University, Shanghai, China

**Keywords:** Insulitis, Panreatic islets, Metformin, Anti-inflammatory effect, STZ, DM

## Abstract

**Background & Aims:**

Metformin is currently the most widely used first-line hypoglycemic agent for diabetes mellitus. Besides glucose-lowering action, there is increasingly interest in the potential anti-inflammatory action of this drug. In the present study, we investigated the actions of metformin on experimental insulitis using STZ-induced diabetic mice.

**Methods:**

Mice with acute diabetes induced by STZ were administered metformin by gavage. Changes of blood glucose and body weight, and the daily amount of food and water intake were measured. Pancreatic tissues were collected for histologic analyses. Pathological assessment and immunohistochemistry analysis were used to determine the effect of metformin on insulitis. Inflammatory cytokines in the pancreas and insulin levels were measured through ELISA analysis.

**Results:**

Metformin significantly reduced blood glucose levels and improved aberrant water intake behavior in experimental diabetic mice. No significant differences were observed in terms of body weight and food intake behavior in metformin-treated animals. In the STZ-induced model of diabetes, we found the appearance of pronounced insulitis. However, metformin administration reduced the severity of insulitis assessed by blind pathological scoring. In addition, metformin treatment improved insulin levels in experimental diabetic mice. ELISA assay revealed decreased levels of inflammatory response marker IL-1*β* and TNF-*α* in the pancreatic tissues following metformin treatment.

**Conclusion:**

Metformin attenuated insulitis in the STZ-induced mice model of diabetes. This islet-protective effect might be partly correlated with the anti-inflammatory action of metformin.

## Introduction

Diabetes mellitus (DM) and its complications constitute a severe public health issue facing modern societies (CDC, 2015). It is generally recognized that there are four major forms of DM, including type 1 and type 2, gestational diabetes and diabetes resulting from other specific factors (CDC, 2015; Jahromi & Eisenbarth, 2007). Type 1 diabetes mellitus (T1DM) is thought originating from an immune-mediated disorder, selectively attacking and making destruction of pancreatic β-cells following inflammatory infiltration of the islets of Langerhans (Campbell-Thompson et al., 2013; Jahromi & Eisenbarth, 2007; Willcox et al., 2009), a pathologic process which was defined as insulitis. Insulitis is pathophysiologically an inflammatory state of the pancreas islets, important parts of the pancreas that are directly responsible for maintaining blood glucose homeostasis (Coppieters et al., 2012; Willcox et al., 2009). Streptozotocin (STZ, 2-deoxy-2 (3-(methyl-3-nitrosoureido) -D-glucopyranose) is commonly used to induce T1DM model (insulin-dependent diabetes mellitus, IDDM), which is mechanistically, at least in part, associated with pronounced insulitis (Szkudelski, 2001). Preclinical studies have suggested that proinflammatory cytokines, such as interleukin (IL)-1β and tumor necrosis factor (TNF)-α, play a critical role in the pathogenesis of T1DM owing to their dramatic effects on pancreatic islets, especially insulin-secreting β-cells (Baumann, Salem & Boehm, 2012; Cnop et al., 2005). Consequently, control of the inflammatory response is a potential therapeutic option for influencing the disease (Baumann, Salem & Boehm, 2012; Citro et al., 2015; Perakakis & Mantzoros, 2016; Waldron-Lynch & Herold, 2011).

Metformin, an oral biguanide class of antihyperglycemic agent, is by far the most widely used glucose-lowering drug for type 2 diabetes mellitus (T2DM) (Ferrannini, 2014). Besides commonly application in T2DM, metformin has been proved beneficial to patients with T1DM, due to improvement of insulin sensitivity (Kaul, Apostolopoulou & Roden, 2015). Recent studies have even suggested the multifunctional profiles of metformin, such as cardiovascular protection, anti-cancer, and anti-inflammatory actions (Foretz et al., 2014; Malinska et al., 2016). Among the multiple actions studied, the anti-inflammatory action of metformin has raised great attention and are thriving for its promising clinical implications (Hattori, Hattori & Hayashi, 2015; Kothari, Galdo & Mathews, 2016; Pernicova & Korbonits, 2014; Saisho, 2015). It has been documented that metformin can activate AMPK/PI_3_K/Akt signaling pathway in human vascular smooth muscle cells, thereby exhibiting anti-inflammatory action via inhibiting NF- κB and subsequent reduction of proinflammatory cytokine generation (Isoda et al., 2006). Moreover, recent clinical studies have suggested that, in patients with impaired glucose tolerance (IGT), metformin treatment helps to down-regulate various proinflmmatory cytokines released from inflammatory cells (Krysiak & Okopien, 2013; Krysiak & Okopien, 2012).

Given the virtue of potential effect against inflammation, we hypothesize that metformin might help attenuate insulitis produced by diabetogenic STZ. We thus, in this paper, sought to determine the effect of metformin on the pancreas injury in STZ-induced diabetic mice model.

## Materials and Methods

### Animals

Male C57BL/6 mice (6∼8 w) were purchased from Sino-British SIPPR/BK Lab Animal Ltd. (Shanghai, China). Mice were maintained in a well-ventilated humidified room (12 h light-dark cycle, 23 ± 2 °C), received water *ad libitum*, and fed standard chow at the Laboratory Animal Science Center of the Second Military Medical University. All the animal experiments were performed in accordance with the National Institutes of Health Guide for the Care and Use of Laboratory Animals. Protocols were approved by the Institutional Animal Care and Use Committee of the Second Military Medical University (SYXK-2012-0003).

### STZ model of diabetes and experimental designs

**Figure 1:**
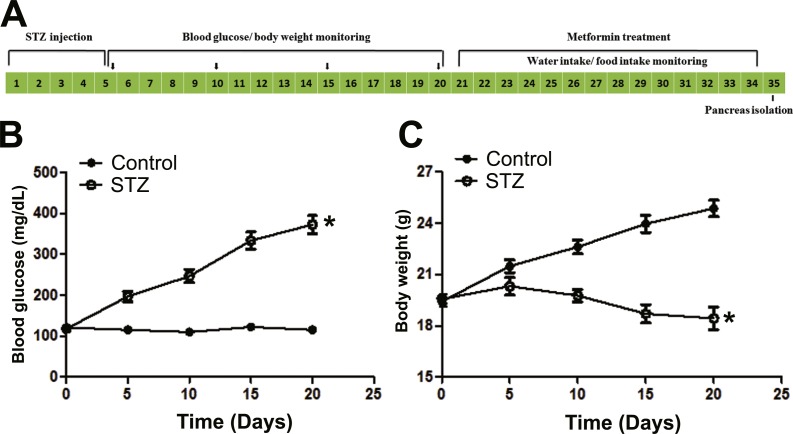
Illustration of experimental design and establishment of STZ induced diabetic mice model. (A) C57BL/6 mice were stimulated with streptozotocin (STZ, 60 mg/kg/d × 5 d, *i.p.*) for five consecutive days, and blood glucose and body weight were monitored every five days until day 20, and then received metformin treatment (250 mg/kg/d × 14 d, *i.g.*) for consecutive 14 days, finally the mice were used for *in vivo* and *ex vivo* studies. Blood glucose concentration (B) and body weight (C) changes in STZ (60 mg/kg/d × 5d, *i.p.*) treated C57BL/6 mice. Diabetic mice (blood glucose level ≥250 mg/dL) were defined 15 days after STZ stimulation. Data are mean ± SEM, (*n* = 8 for all experiments). ^∗^*P* < 0.05 *vs* control.

C57BL/6 mice were given STZ (Amresco, Solon, Ohio, USA) for a consecutive 5-day schedule (at 8∼12 a.m. every day) according to published methods and our previous protocol (Szkudelski, 2001; Yu et al., 2016). Briefly, the compound was dissolved in a citrate buffer (pH 4.5), and injected intraperitoneally (60 mg/kg/d) within 15 min of dissolution. The control group received citrate buffer solution without STZ correspondingly. Three weeks post STZ stimulation, animals with random blood glucose value ≥300 mg/dL were defined as STZ-induced diabetic mice. The mice were then divided into three groups: (1) Control; (2) STZ; and (3) metformin treated (STZ + Met), and housed in groups. The dose of metformin administered to mice in this study was calculated according to clinically relevant human dose based on body surface area. Metformin (250 mg/kg/d; Sigma-Aldrich, St. Louis, MO, USA) dissolved in vehicle solution (5% sodium carboxymethylcellulose, CMC-Na; Sangon Biotech, Shanghai, China) was administered by gavage for 2 weeks (at 2∼4 p.m. every day). Mice in the Control and STZ groups were treated with vehicle correspondingly. On day 34, mice were used for ex vivo studies (Fig. 1A)

### Blood glucose and body weight determinations

Blood samples were collected from the mice tail vein for the determination of blood glucose (at 2∼4 p.m.) according to previously described methods (Li et al., 2016; Yu et al., 2016) using a monitoring system (Major Biosystem, Taipei, Taiwan, China). Changes in the body weight of each group were determined after metformin administration on day 34 at 2∼4 p.m. Also, the daily amount of food and water intake was measured.

### Histological preparation and assessments of pancreatic islets in H&E and immunofluorescence stained sections

C57BL/6 mice were euthanized by cervical dislocation at the end of metformin treatment. Then the pancreas were excised, separated immediately from the fat, cleaned carefully, and blotted dry. The collected pancreata were divided into two parts along the pancreatic duct, either fixed in 10% buffered paraformaldehyde and embedded in paraffin for histological processing, or processed for insulin and cytokines analysis. Thin sections (5 µm) of these embedded samples were cut and stained with hematoxylin and eosin (H&E) for histopathological evaluations. Images were taken at 200× magnifications under an inverted phase-contrast light microscope (Leica Microsystems, Wetzlar, Germany).

For immunohistochemical analysis, insulin in the pancreas islets was detected using a mouse monoclonal antibody (diluted 1:50; BD Bioscience, San Jose, CA, USA) followed by immunofluorescence staining with Cy3-labeled goat anti-mouse IgG antibody (Jackson ImmunoResearch, West Groove, PA, USA). The nuclei were labeled using DAPI (diluted 1:250; Molecular Probes/Invitrogen Life Technologies, Eugene, OR, USA) staining. Images were obtained with a microscope (Leica Microsystems, Wetzlar, Germany) at a magnification of ×200 using identical acquisition settings for each section. The relative intensities of fluorescence, two sections per mouse, were determined using Image-Pro Plus software (Media Cybernetics, Silver Spring, MD, USA).

### Insulitis score

The severity of insulitis was semiquantitatively measured according to previously reported method (Citro et al., 2015) using a scale of 0–4, where 0 referred to no sign of pathological changes in the pancreas islets, 1 referred to peri-insulitis (sign of pathological infiltration changes observed only restricted to the surrounding of islets), 2 referred to mild evidence of insulitis (less than 25% of the islets showed sign of pathological infiltration changes), 3 referred to severe evidence of insulitis (between 25 and 75% of the islets showed sign of pathological infiltration changes), and 4 corresponded to apparent evidence of destructive insulitis (more than 75% of the islets showed sign of pathological infiltration changes). The analysis of islet infiltration was performed by two independent observers who were blinded to the experimental conditions.

### Insulin assay

After all of the final measurements had been obtained, mice were anesthetized with ketamine (100 mg/kg, *i.p.*), and blood samples used for serum insulin measurement were obtained by excising the eyeballs. Those collected pancreatic tissues were homogenized and centrifuged at 3, 200× g for 30 min. The supernatant was collected and stored at −80 °C until further use.

The determination of insulin levels in both serum and pancreatic tissue samples were performed by ELISA according to the manufacturer’s instructions. Kit for estimation of insulin was obtained from the Shibayagi Co., Ltd. (Ishihara, Shibukawa, Gunma, Japan). Briefly, 10 µl of the collected serum and tissue specimens were incubated in wells coated with biotin conjugated monoclonal anti insulin for 2 h at room temperature (RT). After incubation, washing buffer was added to rinse wells, followed by addition of HRP conjugated streptavidin to incubate for 30 min at RT. After washing, the remaining HRP conjugated streptavidin were reacted with substrate chromogen reagent for another 30 min at RT. Reaction was then stopped by addition of acidic solution, and the readings were made on a microplate reader (Bio-Rad, Hercules, CA, USA) at 450 nm. All samples, blanks and standards were analyzed in duplicate.

### Inflammatory cytokines determination

Pancreatic tissue samples were analyzed for proinflammatory cytokines (IL-1β and TNF-α) using ELISA kits (Anogen, Mississauga, Ontario, Canada) according to the manufacturer’s protocols. Estimation of IL-1β and TNF-α were performed using monoclonal anti-mouse IL-1β and TNF-α as primary antibodies. All readings were made on an ELISA Plate Reader (Bio-Rad, Hercules, CA, USA). All samples, standards and blanks were analyzed in duplicate.

### Statistical analyses

Data were expressed as means ± SEM and analyzed by one-way ANOVA followed by Tukey’s test using GraphPad Prism Software (Version 5.01). Statistical significance was set at *P*<0.05.

## Results

### Metformin reduced blood glucose level in STZ-induced diabetic mice

**Figure 2:**
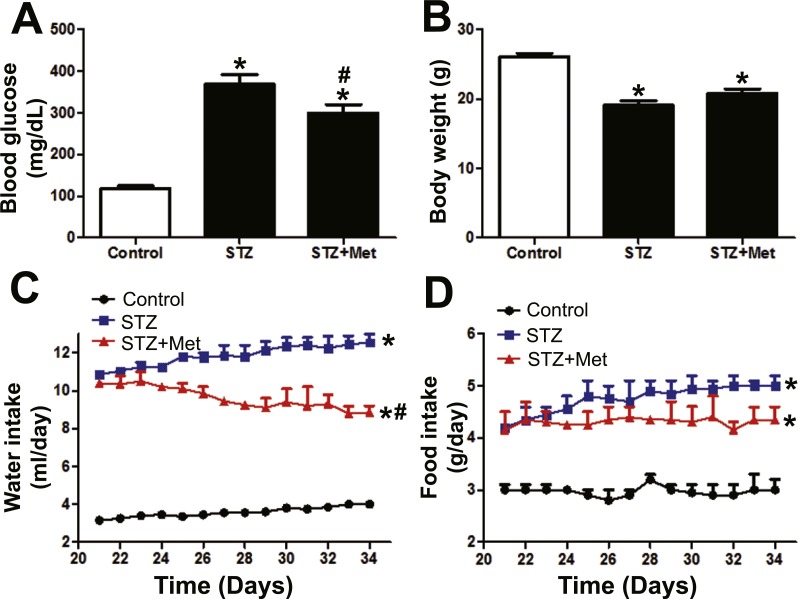
Effects of metformin treatment in STZ-induced diabetic mice on blood glucose, body weight, and feeding behavior. (A) Metformin acutely reduced blood glucose while did not influence body weight (B) on day 34. (C) Significant decreased water intake and (D) slightly improved food intake behavior were observed. Data are mean ± SEM, (*n* = 8 for all experiments). ^∗^*P* < 0.05 *vs* Control, ^#^*P* < 0.05 *vs* STZ.

In Figs. 1B and 1C and Figs. 2A–2B, the plasma glucose levels and body weight changes are shown after injection of STZ or treatment with metformin. Animal glucose levels in the STZ group were significantly elevated. Metformin treatment significantly reduced blood glucose in STZ-induced diabetic mice compared with STZ group (*P* < 0.05). There was no significant difference in the body weight between metformin-treated and SZT group, though the body weight was significantly decreased after injection of STZ (*P* < 0.001).

In addition, improvement of aberrant food and water intake behavior was also observed following metformin treatment (Figs. 2C and 2D). The animals in STZ group evidenced significant increase in both water and food intake after receiving STZ stimulation (Figs. 2C and 2D). The aberrant water intake behavior was significantly improved after metformin treatment compared with vehicle-administered STZ-induced diabetic mice (Fig. 2C), and the increased food intake tended to be lower, but it was not statistically significant (Fig. 2D).

### Metformin increased pancreatic and serum insulin levels in STZ-induced diabetic mice

**Figure 3:**
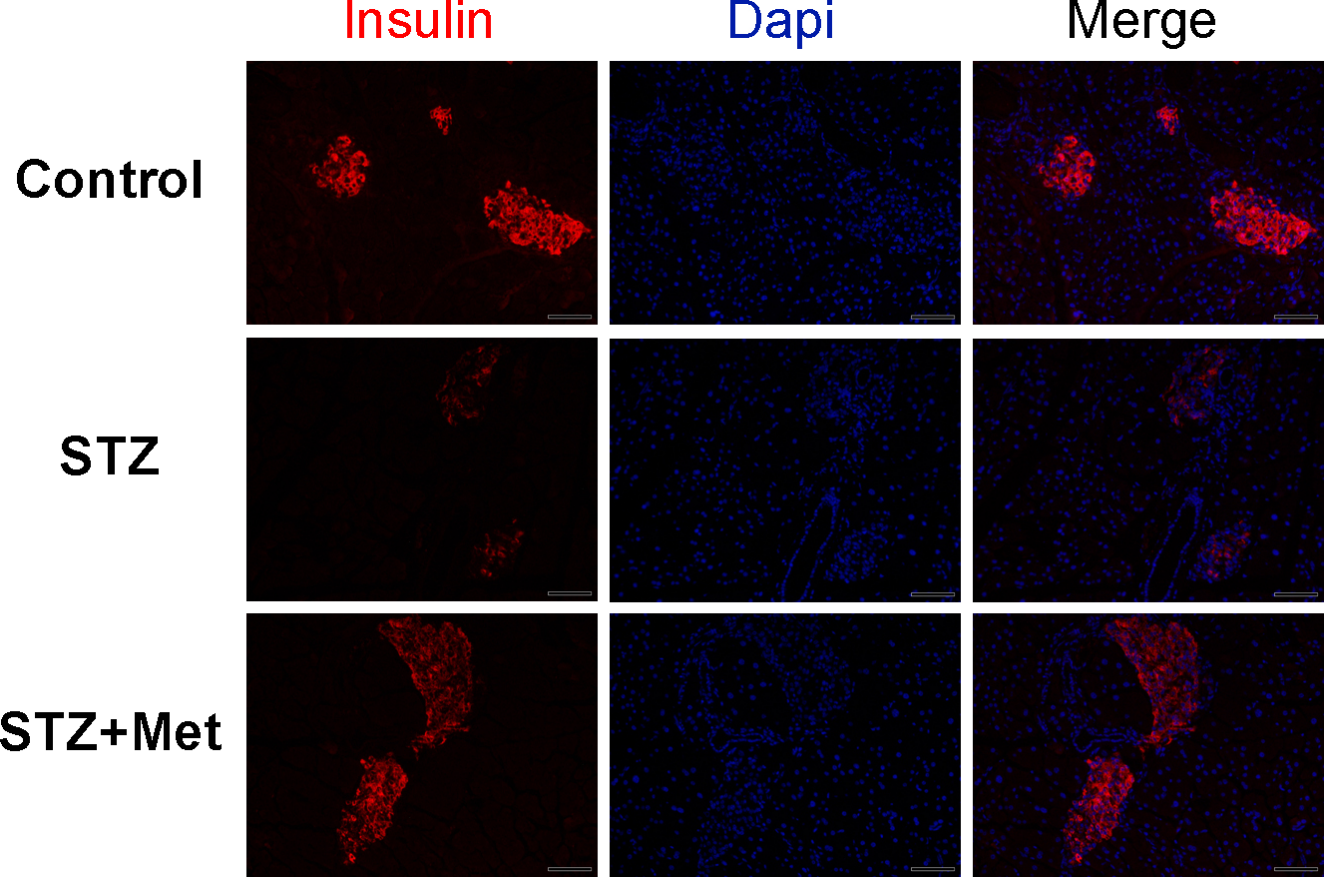
Effect of metformin treatment in STZ-induced diabetic mice on insulin secretion within the pancreatic islet structure. Representative images of the pancreatic tissue sections are stained by immunofluorescence against insulin (insulin staining in red, nuclei staining in blue). All images were taken at ×200 magnification. Scale bars, 50 µm. *N* = 6 for all experiments.

**Figure 4:**
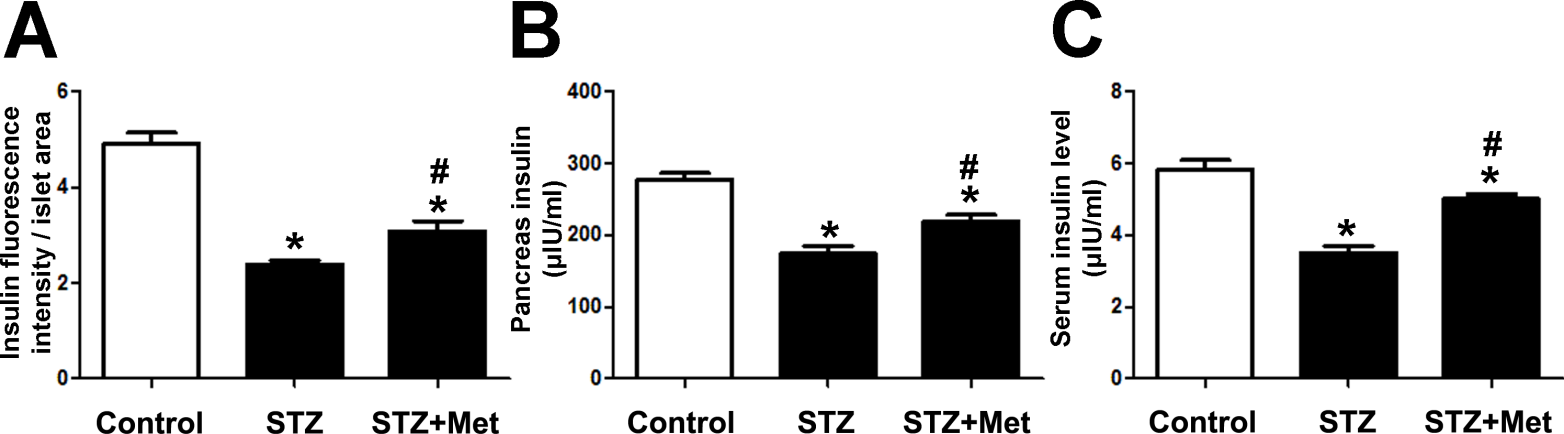
Effects of metformin treatment in STZ-induced diabetic mice on insulin production. (A) Metformin increased insulin intensity within islet structure, and elevated insulin levels in both pancreatic tissues (B) and sera (C). Data are mean ± SEM, ((A) *n* = 6; (B and C) *n* = 12 for all experiments). ^∗^*P* < 0.05 *vs* Control, ^#^*P* < 0.05 *vs* STZ.

To determine the recovery of β-cell function, insulin levels in pancreatic tissues and sera were detected at the end of the study. Significant differences were observed when compared mice stimulated with STZ to mice from the control group (Figs. 3 and 4). However, metformin administration significantly increased insulin secretion of STZ-induced diabetic mice: the insulin levels in either pancreas (*P* < 0.01; Fig. 4B) or serum (*P* < 0.001; Fig. 4C) were significantly improved in mice treated with metformin when compared to those in the STZ group. Also shown in Fig. 4B is ELISA analysis of insulin production in pancreas from different groups. Thus, the application of metformin may provide a significant degree of β-cell protection in STZ-induced diabetes model.

### Metformin attenuated insulitis in STZ-induced diabetic mice

**Figure 5:**
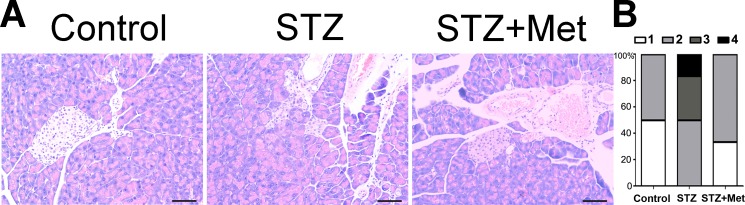
Effect of metformin treatment in STZ-induced diabetic mice on insulitis in pancreatic islets (A) Representative images of the pancreas from different groups were created using hematoxylin and eosin (H & E) stain. The islet tissue is brighter than the pancreatic tissue. All images were taken at ×200 magnification. Scale bars, 50 µm. (B) Insulitis was reduced by metformin administration in diabetic mice model. The percentage of classified islets in each of the five infiltration categories in different groups was as follows: 0, no insulitis; 1, peri-insulitis; 2, mild insulitis (<25% of infiltrated islets); 3, severe insulitis (25–75% of infiltrated islets). *N* = 6 for all experiments.

The administration of metformin has been found to protect against diabetes-related complications in mice/rats (Sartoretto et al., 2005; Yu et al., 2016), when STZ is administered as diabetogenic doses. Figure 5 shows the action of metformin to alleviate the toxic activity of STZ in C57BL/6 mice. In the presence of diabetogenic STZ, the islet integrity became disrupted, with uneven boundaries. However, administration of metformin in STZ-induced diabetic mice preserved islets integrity, with regularly shaped islets observed under microscope (Fig. 5A).

The severity of insulitis was also scored in experimental diabetic mice 2 weeks after treatment to determine the pancreas islet leukocytic infiltration degree. A significant difference was found between the STZ-induced diabetic mice and non-STZ stimulated control mice (Fig. 5). However, the insulitis score in STZ-induced diabetic mice was greatly lowered by metformin administration (Fig. 5B).

### Metformin reduced IL-1β and TNF-α level of pancreatic tissues in STZ-induced diabetic mice

**Figure 6:**
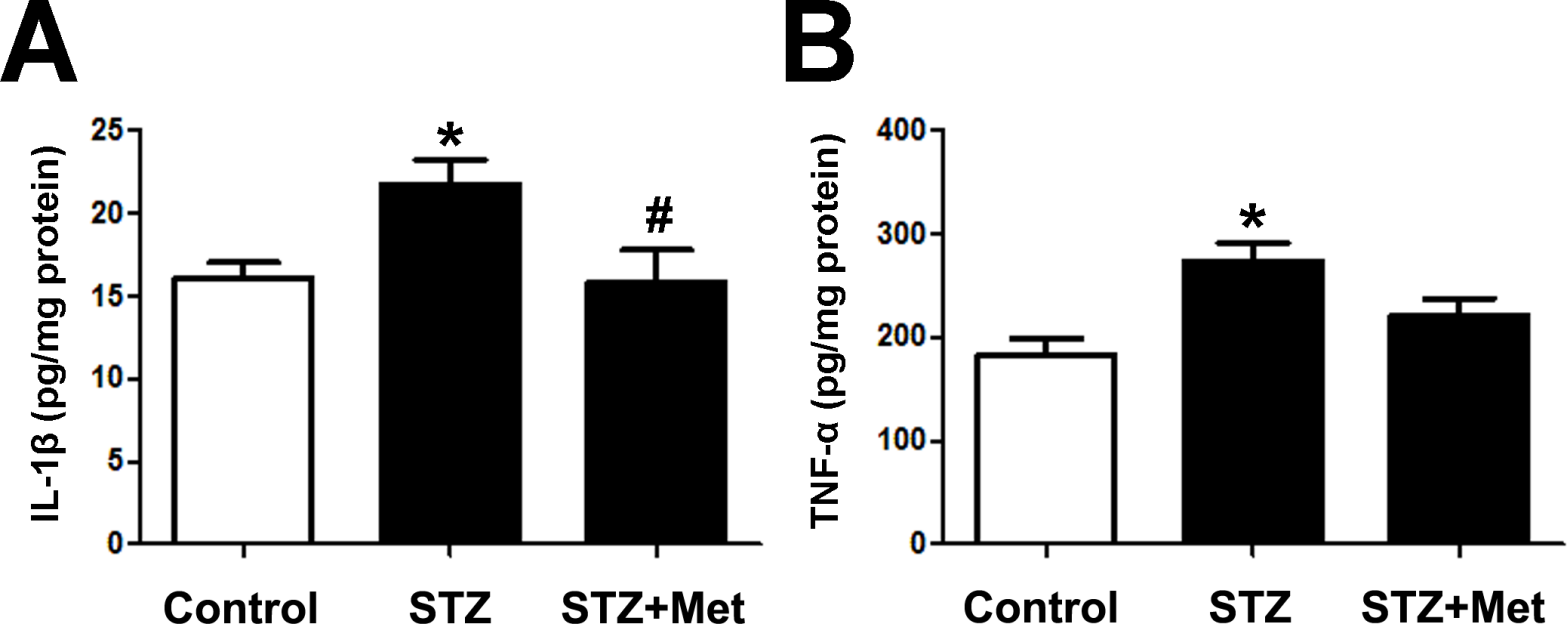
Effects of metformin treatment in STZ-induced diabetic mice on IL-1 *β* and TNF-*α*. (A) Metformin decreased concentrations of IL-1*β* and (B) reduced TNF-*α* concentrations in pancreas. Data are mean ± SEM, ((A) *n* = 7–8; (B) *n* = 7 for all experiments). ^∗^*P* < 0.05 *vs* Control, ^#^*P* < 0.05 *vs* STZ.

Proinflammatory cytokines, such as IL-1β and TNF-α, in pancreas reflects the severity of pancreas islets injury. The administration of metformin has been reported to possess potential anti-inflammatory effects (Saisho, 2015). Therefore, we give this agent to each of the STZ-induced diabetic mice. To determine inflammatory cytokines in the pancreas, tissue IL-1β and TNF-α level were measured at the end of the study. Significant increase of the two cytokines was detected in STZ-induced diabetic mice, compared to those from control group (Fig. 6). Treatment with metformin reduced inflammatory cytokines production in the pancreas of experimental diabetic mice: IL-1β level in mice treated with metformin was similar to that in mice from control group (Fig. 6A); though not statically significant reduced, TNF-α level tended to be lower when compared to STZ-induced diabetic mice (Fig. 6B).

## Discussion

The present study shows the protective effect of metformin against insulitis using STZ-induced diabetic mice model.

Insulitis, defined by the presence of inflammatory infiltration specifically targeting on the pancreas islets, has long been thought correlated with the development of T1DM (Willcox et al., 2009). It has been evidenced that this pathological change can be produced by diabetogenic agent, STZ, commonly used in medical research of experimental T1DM (Rossini et al., 1977). In this study, we found that multiple injections of diabetogenic STZ in C57BL/6 mice produced severe insulitis beyond hyperglycemia and common diabetic symptoms including weight loss, and aberrant food and water intake behaviors.

It has been demonstrated that insulin resistance was associated with a proinflammatory state, which may be mediated by cytokines (Garcia et al., 2010). Advances in basic and experimental science have also elucidated the role of inflammation that contributes to the development of diabetes (Garcia et al., 2010). In the present study, we observed that the pancreas islets were infiltrated with inflammatory cells after STZ injection. Meanwhile, STZ stimulation induced large amount of production of IL-1β and TNF-α, both of which have been suggested to induce islet inflammation and lead to β-cell dysfunction (Eguchi et al., 2012), in the pancreatic tissues. Thus, inflammatory response may probably contribute to the aforementioned pathological damages in STZ-induced experimental diabetic model.

Metformin, the most frequently prescribed hypoglycemic agents, has long been used for antidiabetic treatment (He & Wondisford, 2015; Pernicova & Korbonits, 2014). In accordance with previously and our recent studies (Cheng et al., 2006; Yu et al., 2016), we observed that metformin significantly decreased the blood glucose level in STZ-induced diabetic animal models, while did not influence the body weight. Besides, a significant improvement in the water intake behavior was observed after 2-week oral administration of metformin, accompanied by slight decrease of the daily amount of food intake.

It has been demonstrated that STZ injection causes a decrease of insulin production due to destruction of the β-cells in the pancreas (Citro et al., 2015). Zhou et al. (2015) showed that metformin treatment did not influence insulin levels in either sera or pancreatic tissues when using Kunming mice for STZ induction of diabetic model. Interestingly, we found in the present study that metformin administration significantly increased both serum and pancreatic insulin levels in STZ-induced diabetic C57BL/6 mice. This is probably because the difference in species chosen for STZ induction of diabetic models. Whereas, further efforts are still needed to elucidate this controversial phenomenon.

Preclinical and clinical studies suggested that metformin treatment also exhibited anti-inflammatory action beyond glucose-lowering effect (Hattori, Hattori & Hayashi, 2015; Saisho, 2015). Hyun el al. demonstrated that metformin can reduce the secretion of TNF- α and IL-1β, both of which are proinflammatory cytokines highly correlated with inflammatory response (Hyun et al., 2013; Zhou et al., 2015). However, no convincing data have been presented to date for the effect of metformin on the insulitis associated with diabetes. In fact, we observed in the present *in vivo* study that oral administration of metformin reduced the severity of insulitis in STZ-induced diabetic mice. The histopathologic changes of pancreatic islets were moderately but significantly improved by metformin treatment. Likewise, the islet insulin contents were significantly increased after metformin treatment, as indicated by fluorescence staining. Moreover, our study also showed that the proinflammatory cytokine IL-1β in the pancreatic tissues of diabetic mice was significantly reduced by metformin treatment. TNF-α infiltration also tended to be reduced. Interestingly, we found that metformin was ineffective for protection against islet injury in the genetically modified diabetic model of *db*∕*db* mce (Figure S1 and Data S1). This is probably because of the different underlying mechanisms by which insulitis generated between STZ and *db*∕*db* mice model. Further evaluation of metformin treating diabetes-associated insulitis is needed using other clinically relevant models, such as *ob/ob* mice and high-fat diet feeding mice.

In conclusion, we herein show the proof that metformin protected against insulitis associated with STZ-induced DM. The evidence of reduction of proinflammatory cytokines, such as IL-1β and TNF-α, in the pancreatic tissues suggests that anti-inflammatory actions of metformin might partly contribute to amelioration of insulitis. This study raises the possibility of using metformin to alleviate insulitis in diabetic individuals, suggesting the clinical advantage of metformin during anti-diabetic treatment. Further investigations aimed to delineate the mechanisms of metformin on insulitis are indicated.

##  Supplemental Information

10.7717/peerj.3155/supp-1 Figure S1 Effect of metformin on pancreatic islet in db/db mice. (A) Representative images of the pancreas were created using hematoxylin and eosin (H and E) staining. There were no significant histological changes in pancreatic islets after metformin treatment (200×, Scale bar = 50 µm). ELISA analysis of insulin levels in pancreatic tissues (B) and serum (C). There were no statistical significance in both pancreatic insulin level and serum insulin level between the two groups (Mean ±SEM, n=8 per group).

10.7717/peerj.3155/supp-2 Data S1 Supplemental data

10.7717/peerj.3155/supp-3 Data S2 Raw data

## Additional Information and Declarations

### Data Availability

The following information was supplied regarding data availability: The raw data has been supplied as a  Supplementary File.
