# Supplementary figures and images for: Metformin ameliorates insulitis in STZ-induced diabetic mice
Source: PeerJ. 2017 Apr 13;5:e3155. doi: 10.7717/peerj.3155 (PMC5399881; doi:10.7717/peerj.3155)

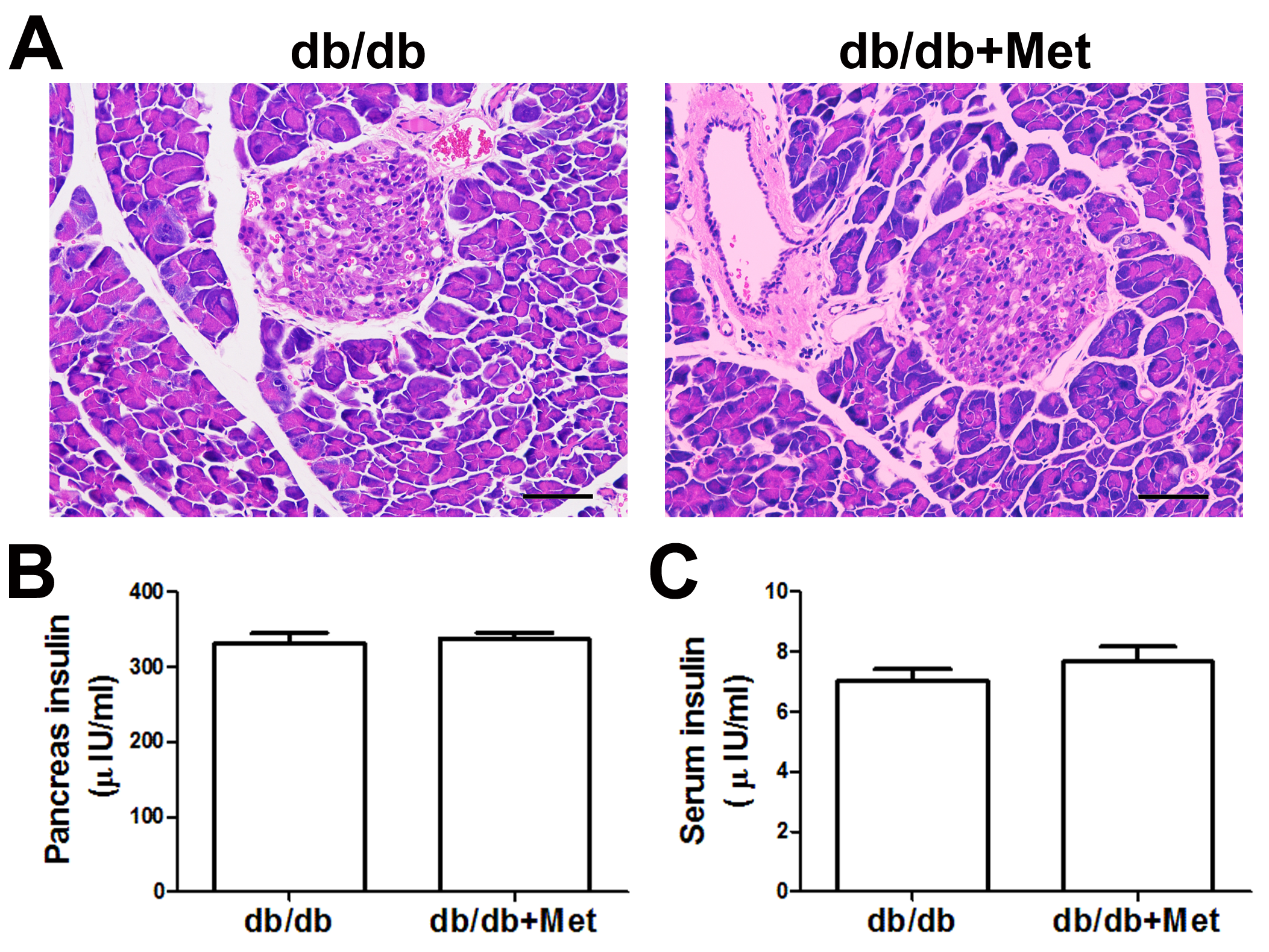

Supplement: Figure S1 — (A) Representative images of the pancreas were created using hematoxylin and eosin (H and E) staining. There were no significant histological changes in pancreatic islets after metformin treatment (200×, Scale bar = 50 µm). ELISA analysis of insulin levels in pancreatic tissues (B) and serum (C). There were no statistical significance in both pancreatic insulin level and serum insulin level between the two groups (Mean ±SEM, n=8 per group). [file peerj-05-3155-s001.png]
